# Severe COVID-19 Patients Show an Increase in Soluble TNFR1 and ADAM17, with a Relationship to Mortality

**DOI:** 10.3390/ijms22168423

**Published:** 2021-08-05

**Authors:** Yadira Palacios, Andy Ruiz, Lucero A. Ramón-Luing, Ranferi Ocaña-Guzman, Omar Barreto-Rodriguez, Anahí Sánchez-Monciváis, Brenda Tecuatzi-Cadena, Ana G. Regalado-García, Rey David Pineda-Gudiño, Alicia García-Martínez, Fortunato Juárez-Hernández, Juan Pablo Farias-Contreras, Ingrid Fricke-Galindo, Gloria Pérez-Rubio, Ramcés Falfán-Valencia, Ivette Buendia-Roldan, Karen Medina-Quero, Leslie Chavez-Galan

**Affiliations:** 1 Instituto Nacional de Enfermedades Respiratorias Ismael Cosío Villegas, Mexico City 14080, Mexico; yadpal@gmail.com (Y.P.); andy.ruiz@iner.gob.mx (A.R.); ramonluing@yahoo.com.mx (L.A.R.-L.); aro-cana@iner.gob.mx (R.O.-G.); omar_barreto10@hotmail.com (O.B.-R.); drjuarez.radiologo@gmail.com (F.J.-H.); juanpa_000@hotmail.com (J.P.F.-C.); ingrid_fg@yahoo.com.mx (I.F.-G.); glofos@yahoo.com.mx (G.P.-R.); rfalfanv@iner.gob.mx (R.F.-V.); ivettebu@yahoo.com.mx (I.B.-R.); 2 Laboratorio de Inmunología, Escuela Militar de Graduados de Sanidad, SEDENA, Mexico City 11200, Mexico; amoncivais11@gmail.com (A.S.-M.); tecuatzi.brenda@gmail.com (B.T.-C.); ag.regaladogarcia@gmail.com (A.G.R.-G.); kmq.kmq5@gmail.com (K.M.-Q.); 3 Hospital Central Militar, SEDENA, Mexico City 11200, Mexico; reydavidneumo@gmail.com (R.D.P.-G.); atlscooper@hotmail.com (A.G.-M.)

**Keywords:** COVID-19, solTNF, solTNFR1, solTNFR2, ADAM17, severity

## Abstract

Overproduction of inflammatory cytokines is a keystone event in COVID-19 pathogenesis; TNF and its receptors (TNFR1 and TNFR2) are critical pro-inflammatory molecules. ADAM17 releases the soluble (sol) forms of TNF, TNFR1, and TNFR2. This study evaluated TNF, TNFRs, and ADAM17 at the protein, transcriptional, and gene levels in COVID-19 patients with different levels of disease severity. In total, 102 patients were divided into mild, moderate, and severe condition groups. A group of healthy donors (HD; *n* = 25) was included. Our data showed that solTNFR1 and solTNFR2 were elevated among the COVID-19 patients (*p* < 0.0001), without increasing the transcriptional level. Only solTNFR1 was higher in the severe group as compared to the mildly ill (*p* < 0.01), and the level was higher in COVID-19 patients who died than those that survived (*p* < 0.0001). The solTNFR1 level had a discrete negative correlation with C-reactive protein (*p* = 0.006, Rho = −0.33). The solADAM17 level was higher in severe as compared to mild disease conditions (*p* < 0.01), as well as in COVID-19 patients who died as compared to those that survived (*p* < 0.001). Additionally, a potential association between polymorphism *TNFRSF1A*:rs767455 and a severe degree of disease was suggested. These data suggest that solTNFR1 and solADAM17 are increased in severe conditions. solTNFR1 should be considered a potential target in the development of new therapeutic options.

## 1. Introduction

Coronavirus disease 2019 (COVID-19) is an infectious disease caused by severe acute respiratory syndrome coronavirus 2 (SARS-CoV-2) [1]. COVID-19 patients show a broad spectrum of symptoms, ranging from common clinical manifestations such as fever, cough, and fatigue, to the development of pneumonia and multi-organ dysfunction, which appears to be a leading cause of death in COVID-19 patients [2]. By 10 June 2021, around 173,989,093 confirmed cases were estimated, including 3,756,947 deaths worldwide [3]. This epidemiological hallmark indicates that urgent need to focus our efforts on the search for therapeutic options and the identification of potential target biomarkers for severity and mortality.

Several risk factors are associated with a progressive SARS-CoV-2 infection, and the uncontrolled overproduction of inflammatory cytokines is a keystone event that contributes significantly to pathogenesis. Some of the reported pro-inflammatory cytokines are interleukin (IL)-6, IL-8, and IL-1β, and tumor necrosis factor (TNF) [4,5]. Here, it is essential to note that some research groups suggest that the baseline level of TNF is not distinct between mildly, severely, and critically ill patients with COVID-19, but others propose that the TNF level is higher in severely ill COVID-19 patients [6,7,8]. This discrepancy is probably because diverse cells subpopulations regulate TNF production. In this regard, Su et al. [9] reported the increase of a specific cluster of monocytes that is reminiscent of an immunoparalysis condition, favoring the decrease of TNF transcripts. Thus, the TNF level requires an integrated view of immune cell subpopulations.

TNF is a critical pro-inflammatory cytokine produced by diverse cell subpopulations. Initially, it is synthesized as a transmembrane molecule (tmTNF), and under activation stimuli, tmTNF is released to the soluble TNF (solTNF) by the action of the A disintegrin and metalloprotease 17 (ADAM17, also called TACE). Both TNF forms mediate diverse functions through interaction with its receptors, TNFR1 and TNFR2, which also are shed by the ADAM17 proteolytic action. Moreover, ADAM17 also mediates the shedding of angiotensin-converting enzyme 2 (ACE2), a receptor for SARS-CoV-2 [10,11].

In the context of SARS-CoV-2 infection, it has not been clarified if the virus encodes soluble TNFRs analogs (typically termed as TNF decoy receptors), which may affect TNF/TNFRs axis signaling. However, reports have shown that the large DNA viruses (poxviruses and herpesviruses) encode this type of solTNFR2 homolog, and these can modify the cytokine response by inhibiting TNF. Anti-TNF strategies allow the progression of viral infection [12,13,14].

For more than 20 years, anti-TNF therapy has efficiently been used to treat autoimmune inflammatory diseases and viral lung diseases [15]. Several commercial inhibitors of the TNF pathway are available, such as infliximab, adalimumab, certolizumab, golimumab, and etanercept, which block TNF and the development of selective TNFR1 and TNFR2 inhibitors. Recently, Feldmann et al. [16] suggested that evidence supports clinical trials of anti-TNF therapy in patients with COVID-19. However, although anti-TNF therapy is used in many inflammatory diseases, for the context of COVID-19 the findings related to TNF level are unclear, and the information regarding TNFR levels among the patients of COVID-19 is scarce, indicating that more studies are necessary to consider anti-TNF therapy in COVID-19 patients.

Thus, this study aims to evaluate TNF/TNFR molecule levels in mildly, severely, and critically ill patients suffering from COVID-19 to provide evidence about their behavior during different stages of severity. Furthermore, this information is relevant in order to study the possibility of establishing anti-TNF therapy in COVID-19 patients.

## 2. Results

### 2.1. Baseline Characteristics of COVID-19 Patients

The study included a total of 102 patients diagnosed with pneumonia due to COVID-19, as evidenced by real-time reverse transcriptase tests (RT-PCR) for SARS-CoV-2 in a nasopharyngeal swab. Patients were recruited from the National Institute of Respiratory Diseases (INER) and the Central Military Hospital (HCM). Table 1 shows the demographic characteristics of the study population. COVID-19 patient groups had an age range between 44 and 53 years. The male gender portion is directly proportional to disease severity; males formed 58.8% of the mild group, 67.7% of the moderate group, and 72.2% of the severe group. In concordance with other COVID-19 cohorts [17,18], comorbidities such as smoking, diabetes mellitus, and systemic hypertension prevailed in the severe group; however, body mass index was increased in the overall studied population regarding overweight and obesity, reflecting a public health problem in Mexico [19].

COVID-19 patients groups displayed diverse clinical manifestations, but all the patients showed at least two of the commonly reported symptoms, and the severe COVID-19 group showed high levels of breathing frequency, low oxygen saturation (Table 2), and high levels of C-reactive protein (CRP) and fibrinogen (Table 3).

### 2.2. Survival of COVID-19 Patients Is Related to Illness Severity and Hospitalization Days

**Figure 1:**
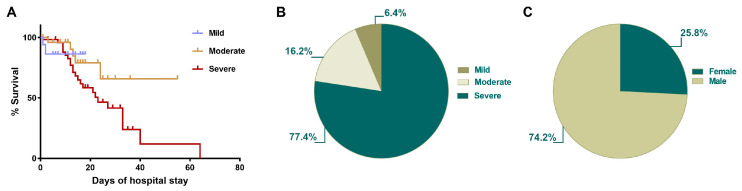
Survival of patients with SARS-CoV-2 infection decreased according to illness severity and hospital stay. (**A**) Kaplan–Meier survival curve in COVID-19 patients. The severe condition group had a hospital stay of 1–64 days (mean of 14.9 days), patients with a moderate illness had a hospital stay between 1 and 55 days (mean of 14.2 days), and the mild group had a hospital stay between 1 and 18 days (mean of 7.9 days. (**B**) Total cohort mortality was 30.4%, with the severe group tallying 77.4%, while moderate group represented 16.2% and the mild group 6.4% of deaths. (**C**) The higher death rate occurred in the male population with a severe classification; 31 patients died: 23 (74.2%) were male, and 8 (25.8%) were female.

A comparative analysis between survival and the clinical course in-hospital stay was performed (Figure 1). Our data showed that those with severe COVID-19 had the most extensive hospitalized period, with a mean of 14.9 days (1–64 days, min–max), whereas the mean of hospitalized days in the moderate group was 14.2 (1–55 days, min–max), and for the mild group the mean was 7.9 (1–18 days, min–max) (Figure 1A). Furthermore, the total cohort mortality was 30.4%, with this being predominant in the severe group (77.4% of deaths), whereas the moderate group represented 16.2% of deaths, and the mild group 6.4% of deaths (Figure 1B). Finally, in concordance with previous reports [20], the male population was the one in which mortality predominated (74.2%) (Figure 1C).

### 2.3. Soluble Levels of TNFR1 and TNFR2 Are Increased in the Serum of COVID-19 Patients

**Figure 2:**
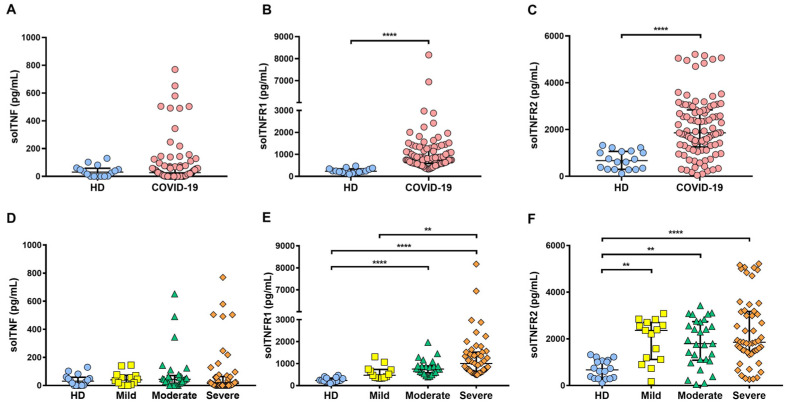
solTNFR1 and solTNFR2 are increased in COVID-19 patients. Soluble forms of TNF, TNFR1, and TNFR2 were quantified in the sera of COVID-19 patients and healthy donors (HD) by ELISA sandwich assay. A general comparison of solTNF, solTNFR1, and solTNFR2 levels between HD and COVID-19 was performed (**A**–**C**, respectively). In addition, a more detailed analysis between HD and groups of COVID-19 (mild, moderate, and severe) was performed to confirm if solTNF, solTNFR1, and solTNFR2 levels were different (**D**–**F**, respectively). Data are presented as median with interquartile range (solTNF: HD *n* = 16, mild *n* = 14, moderate *n* = 29, and severe *n* = 50; solTNFR1: HD *n* = 16, mild *n* = 13, moderate *n* = 24, and severe *n* = 49; solTNFR2: HD *n* = 18, mild *n* = 15, moderate *n* = 29, and severe *n* = 53), **** *p* < 0.0001, ** *p* < 0.01.

To clarify if COVID-19 patients have altered levels of the TNF pathway molecules, solTNF, solTNFR1, and solTNFR2 levels were examined by enzyme-linked immunosorbent assay (ELISA) (Figure 2). First, we carried out a general comparison between the healthy donor (HD) and COVID-19 groups, and our data revealed that the solTNF level did not exhibit a significant difference; however, solTNFR1 and solTNR2 levels were statistically different (higher, in the case) in the case of COVID-19 as compared to HD (*p* < 0.0001) (Figure 2A–C).

solTNF, solTNFR1, and solTNFR2 levels were compared among COVID-19 groups. Although some patients of the moderate and severe groups showed high solTNF levels, there were no statistically significant differences (*p* = 0.55) (Figure 2D). The solTNFR1 level was not different between mild group versus HD, but in contrast, moderate and severe COVID-19 patients had a high level of solTNFR1 compared to HD (*p* < 0.0001). Interestingly, severe COVID-19 patients had higher solTNFR1 levels than those in mild condition (Figure 2E). Finally, the solTNFR2 level was higher in each status of COVID-19 as compared to the HD group (mild and moderate *p* < 0.01, severe *p* < 0.0001), but there were no differences between the COVID-19 status groups (Figure 2F). In summary, our results suggest that solTNF level is not increased during COVID-19, but solTNFRs levels are increased, and specifically, there were differences in solTNFR1 between mild and severe COVID-19 groups.

### 2.4. TNFR1 and TNFR2 Are Not Increased at the Transcriptional Level in COVID-19 Patients, while ADAM17 Is Increased

**Figure 3:**
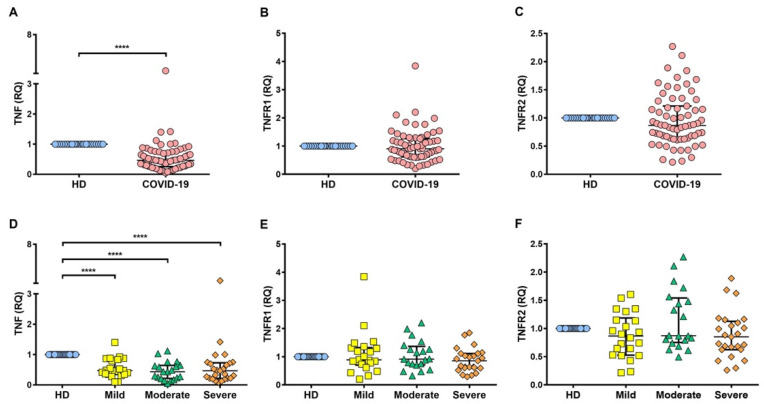
TNF, TNFR1, and TNFR2 are not increased at the transcriptional level in COVID-19 patients. The relative quantification (RQ) of TNF, TNFR1, and TNFR2 transcripts was evaluated by qRT-PCR in PBMCs of healthy donors (HD) and COVID-19 patients. A comparison between HD and COVID-19 provides evidence that for TNF, TNFR1, and TNFR2, the RQ was not increased in COVID-19 patients (**A**–**C**, respectively). There was a significant difference in the decrement in TNF. Between COVID-19 groups (mild, moderate, and severe) (**D**), regarding receptors there were no differences (**E**,**F**). The Ct values for each gene were normalized to the endogenous control gene 18S rRNA, and the relative expression of transcripts was quantified by the ΔΔCT method. Data are presented as median with interquartile range (HD *n* = 10, mild *n* = 22, moderate *n* = 21, and severe *n* = 25). **** *p* < 0.0001.

To verify that the increment detected at the protein level is presented at the transcriptional level, the relative gene expression of TNF, TNFR1, and TNFR2 was evaluated (Figure 3). TNF at the transcriptional level was decreased in COVID-19 patients as compared to HD (*p* < 0.0001) (Figure 3A), and this mRNA level was maintained for each type of COVID-19 severity (*p* < 0.0001) (Figure 3D). Surprisingly, TNFR1 and TNFR2 were not increased in COVID-19 patients as compared to HD (Figure 3B,C). This behavior was maintained even when comparing COVID-19 status (Figure 3E,F).

**Figure 4:**
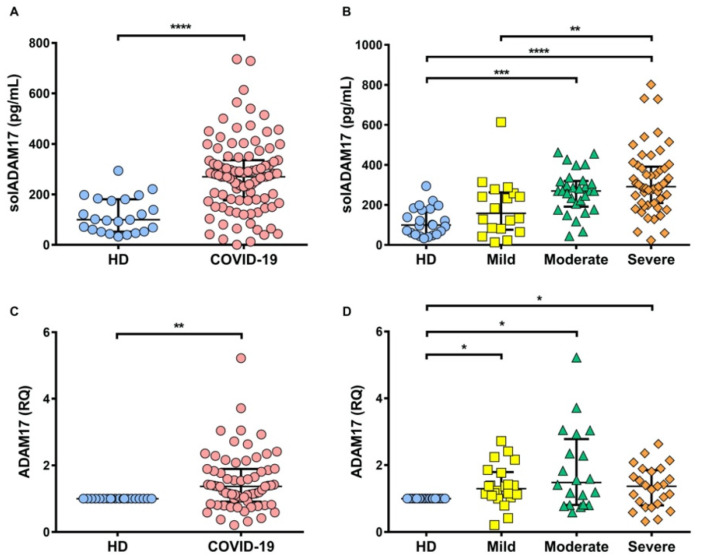
ADAM17 is increased at the protein and transcriptional levels for COVID-19 patients. ELISA assay sandwich was performed comparing healthy donors (HD) against the COVID-19 group (**A**) and comparing COVID-19 groups (HD *n* = 23, mild *n* = 19, moderate *n* = 29, and severe *n* = 53). (**B**) in both cases, solADAM17 was increased. Relative quantification (RQ) of ADAM17 was evaluated by qRT-PCR from PBMC of HD and COVID-19 patients. A comparison against HD (**C**) and between COVID-19 groups (**D**) showed that ADAM17 gene expression was increased (HD *n* = 10, mild *n* = 22, moderate *n* = 20, and severe *n* = 25). The Ct values of ADAM17 were normalized with the endogenous control genes 18S rRNA, and the relative expression of transcripts was quantified by the ΔΔCT method. Data are presented as median with interquartile range, **** *p* < 0.0001, *** *p* < 0.001, ** *p* < 0.01, and * *p* < 0.05.

ADAM17 is responsible for delivering the soluble forms of TNF, TNFR1, and TNFR2 [21,22]; we hypothesized COVID-19 patients had increased ADAM17, explaining why TNFRs levels increased. ADAM17 was evaluated at transcriptional and protein levels. Our results confirm that compared to HD, COVID-19 patients have increased ADAM17 at both proteins (*p* < 0.0001) and transcriptional levels (*p* < 0.01) (Figure 4A,C). ADAM17 level is maintained high at the protein and transcriptional level for each status of COVID-19, but solADAM17 level is higher in severely as compared to mildly ill COVID-19 patients (Figure 4B,D).

### 2.5. solTIM3 Level, but Not TGF-β, Is Increased in the Serum of COVID-19 Patients

ADAM17 sheds several proteins on the cell membrane to deliver their soluble forms, including ACE2 that is the receptor for SARS-CoV2, T cell immunoglobulin and mucin domain 3 (TIM3), and transforming growth factor-beta (TGF-β) [23,24].

**Figure 5:**
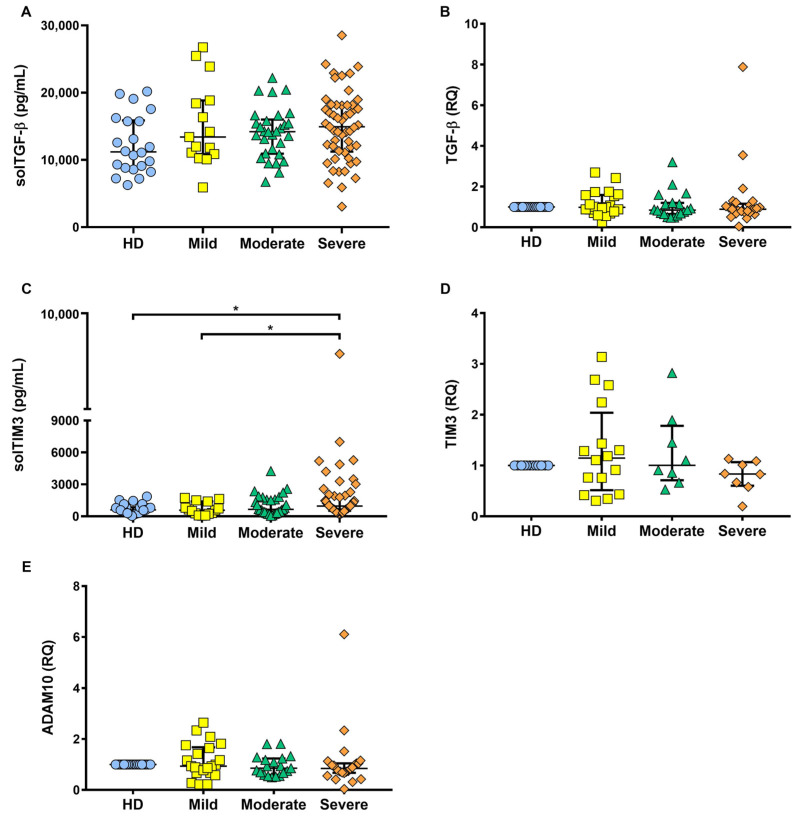
solTIM3 is increased in COVID-19 patients but not TGF-β. The soluble forms of TGF-β and TIM3 were measured by ELISA sandwich assay (**A**,**C**, respectively). As compared to the healthy donors (HD), only solTIM3 was significantly increased in COVID-19 patients. At the transcriptional level as evaluated by qRT-PCR, no differences were found in the relative quantification (RQ) of TGF-β, TIM3, and ADAM10 (**B**,**D**,**E**, respectively) between HD and the group of COVID-19 patients (mild, moderate, and severe); (solTGF-β: HD *n* = 22, mild *n* = 15, moderate *n* = 30, and severe *n* = 53; solTIM3: HD *n* = 23, mild *n* = 15, moderate *n* = 31, and severe *n* = 54). Ct values were normalized to the endogenous control gene 18S rRNA; the relative expression of transcripts was quantified by the ΔΔCT method. Data are presented as median with interquartile range (TGF-β and ADAM10: HD *n* = 10, mild *n* = 22, moderate *n* = 19, and severe *n* = 25; TIM3: HD *n* = 10, mild *n* = 16, moderate *n* = 8, and severe *n* = 8), * *p* < 0.05.

To confirm whether ADAM17 was shedding, the molecules preferably related to the TNF pathway, TIM3, and TGF-β, were measured at the transcriptional and protein levels (Figure 5).

Results revealed that TGF-β was not increased in any COVID-19 status at the protein and transcriptional level (Figure 5A,B). Soluble TIM3 (solTIM3) level was increased in severely ill COVID-19 patients, with statistically significant differences as compared to both HD and mildly ill COVID-19 patients (*p* < 0.05) (Figure 5C). However, at the transcriptional level, TIM3 was not different among the COVID-19 groups (Figure 5D). Furthermore, considering that TIM3 is cleaved in both ADAM17 and a disintegrin and metalloproteinase domain-containing protein 10 (ADAM10) [25], we measured ADAM10 (transcriptional level), but our data showed that ADAM10 was not increased in any of the COVID-19 patient groups (Figure 5E). These results reinforce our hypothesis that the shedding process plays a significant role in delivering protein soluble forms, because, similar to TNFR1, TIM3 is not increased at the transcriptional level, but the soluble form is increased.

### 2.6. A High Level of solTNFR1 Is Associated with COVID-19 Deaths and with a Low Level of C-Reactive Protein

**Figure 6:**
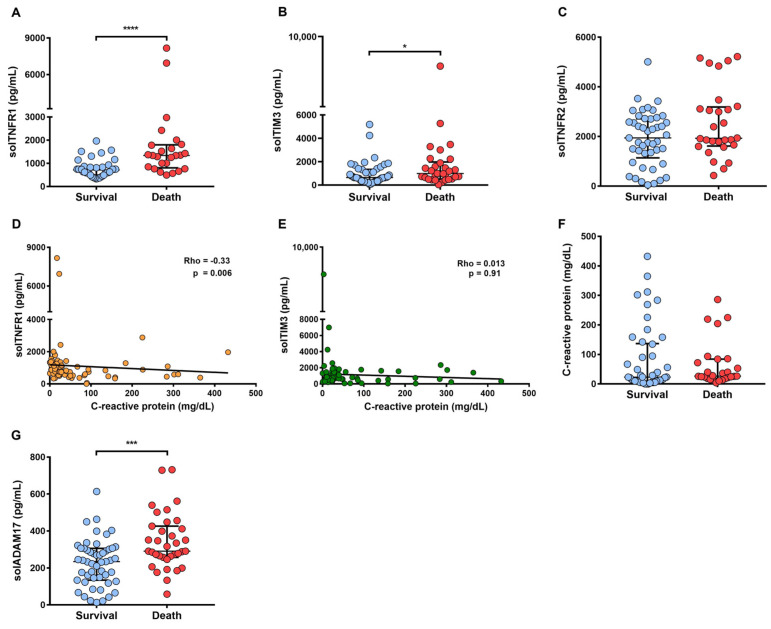
Association of solTNFR1 with death and the negative correlation with C-reactive protein (CRP) in COVID-19. Comparison between survival and death regarding solTNFR1, solTIM3, solTNFR2, CRP, and solADAM17 (**A**–**C**,**F**,**G**, respectively). Data are presented as medians with interquartile ranges (solTNFR1: survival *n* = 39, death *n* = 25; solTIM3: survival *n* = 46, death *n* = 30; solTNFR2: survival *n* = 45, death *n* = 28; CRP: survival *n* = 46, death *n* = 27; solADAM17: survival *n* = 52, death *n* = 37). Spearman’s correlations between solTNFR1 and solTIM3 with CRP were examined (**D**,**E**, respectively). **** *p* < 0.0001, *** *p* < 0.001, * *p* < 0.05.

Since solTNFR1 and solTIM3 levels are higher in severely ill as compared to mildly ill COVID-19 patients and both molecules are delivered by the effect of the same shedder, we analyzed the levels of these molecules among patients who survived as compared to the dead subjects (Figure 6). In addition, the evaluation of solTNFR2 was included because levels did not differ among the COVID-19 groups, but they did differ when comparing HD and COVID-19 patients.

Our data show that the COVID-19 death patients had high levels of solTNFR1, solTIM3, and solADAM17 as compared to COVID-19 patients who survived (*p* < 0.0001, *p* < 0.05, *p* < 0.001 respectively) (Figure 6A,B,G), and the solTNFR2 level did not differ (Figure 6C).

To evaluate if those molecules were associated with a classically pro-inflammatory clinical parameter such as CRP, a Spearman correlation test was performed to confirm this association. We found that a high level of solTNFR1 correlated with a low level of CRP (*p* = 0.006, Rho = −0.33) (Figure 6D), which was not observed in solTIM3 (*p* = 0.913, Rho = 0.01) (Figure 6E). It is important to note that the CRP level did not differ for COVID-19 patients who died as compared to those that survived (*p* = 0.31) (Figure 6F). Thus, COVID-19 patients who died had a high level of solTNFR1, with a negative correlation with CRP; these data suggested that other pro-inflammatory mechanisms (independent of the classical CRP) could be activated in severe COVID-19 patients.

### 2.7. Genetic Variants in TNF, TNFRSF1A, and TNFRSF1B

We assessed the allele and genotype frequencies of the *TNF* gene and the receptors *TNFRSF1A* and *TNFRSF1B*, which are included in the supplemental material (Table S1). Although no significant differences were observed among groups, a trend *p*-value was observed when the C allele frequency of rs767455 *TNFRSF1A* variant was compared with regard to patients with severe COVID-19 and those included in the mild disease group (45.5% vs. 25.0%, *p* = 0.0583). This was also observed for the CC and TC genotype frequencies for the same variant. Moreover, in the dominant model analysis, the frequency of rs1800693 TC + CC genotypes was also marginally significant between the severely and mildly ill patients (72.7% vs. 44.4%, *p* = 0.069) (Table S2).

## 3. Discussion

A common clinical complication associated with COVID-19 is acute respiratory distress syndrome (ARDS) attributed to a cytokine storm of IL-1β, IL-6, IFN-γ, and TNF, among others. Several treatment schemes have been suggested to prevent ARDS and death in patients with COVID-19. However, the diversity of therapeutic approaches with heterogeneous results forces targeting strategies aimed at deregulated targets [26,27]. Currently, various treatment options have been explored for COVID-19, for instance monoclonal antibodies (bamlanivimab plus etesevimab, casirivimab plus imdevimab, tocilizumab), antivirals (remdesivir), dexamethasone, prednisone, methylprednisolone, hydrocortisone, and molecule-specific inhibitors (baricitinib). Although anti-TNF treatment was suggested as a potential treatment for COVID-19, insufficient data exists to support the use of anti-TNF therapy because reports indicate diverse efficiency [16,26,28,29]. Further research is required focusing on the development of immunomodulators.

This study evaluated a cohort of 102 COVID-19 patients with different degrees of severity. Two health institutions provided the patients, and systematically high solTNFR1 and soluble ADAM17 levels were observed in the severely ill COVID-19 group and patients who died. Interestingly, high solTNFR1 levels are associated with low CRP; these results bring up a new debate for the study of those inflammatory mechanisms regulated by TNFR1 or TNFR2 pathways, such as those related to the mitochondria or endoplasmic reticule damage.

In concordance with our study, Mortaz et al. recently reported that solTNFR1 is associated with mortality in severe cases of COVID-19 [30]. Previously, Bowman et al. [31] reported that both solTNFR1 and solTNFR2 are higher in critical patients who died than in critical patients who recovered. Although our cohort confirmed elevated levels of both soluble TNF receptors in COVID-19 patients, we did not find a higher level of solTNFR2 in patients who died or in patients with a severe condition. Thus, in accordance with the report of Mortaz, we suggest that a high solTNFR1 level is associated with severe COVID-19 patients and, in addition, our data support the evidence that high solTNFR1 levels are related to an increase of solADAM17. Together, these results suggest that the excess of solTNFR1 could mediate interactions with cells and active other non-classical inflammatory mechanisms that are not CRP-dependent because, as is discussed below, TNFR1 signaling mediates inflammation and cell death.

TNF plays a role in the hyper-inflammation during SARS-CoV-2 infection, but its function cannot be separated from its receptors or shedder; results obtained regarding transcriptional and TNFRs protein levels are contradictory. The increased levels of soluble TNFRs are probably a consequence of the high level of ADAM17. Thus, increased TNFR levels are not the result of the excessive production by cells but rather an increase in ADAM17. This is one of the most important reasons suggesting that trials for anti-TNF therapy should include the measure of TNF and its receptors to direct the efforts in developing the best alternative schemes of treatment for COVID-19.

Recently, Saleh et al. [32] reported that the A allele of the *TNF* G-308 A promoter variant (rs1800629) is associated with a more aggressive COVID-19 pattern, and authors suggested that the use of anti-TNF therapy may be promising in those patients. Although this variant has also been related to differences in protein expression in other lung diseases [33,34], unfortunately the G-308/severe COVID-19 association failed to be validated in larger studies in other populations [35,36]. We found no evidence of an association between *TNF*, *TNFRSF1A*, and *TNFRSF1B* gene variants with regard to COVID-19 severity, but we did see a trend of *TNFRSF1A* polymorphism, suggesting that with greater power we could have produced more significant findings. These results should be deeply studied to clarify the impact of the use of anti-TNF therapy.

Reports have been demonstrated that TNFR1 signaling mediated the initiation of molecular pathways to lead to inflammation and cell death [37,38]. Therefore, it is not surprising that other molecules associated with a damage-associated molecular pattern like high mobility group box 1 (HMGB1) are elevated in severe COVID-19 patients. Consequently, HMGB1 inhibitors are suggested as drug candidates for the treatment of SARS-CoV-2 infection [39]. PANoptosis is defined as an inflammatory programmed cell death activated by specific triggers, and TNFR1 is associated with the activation of different types of cell death (pyroptosis, apoptosis, necroptosis). Interestingly, it has been reported that the combined effect of TNF and IFN-γ induces inflammatory cell death that favors a lethal cytokine shock, and the authors suggested that the use of neutralizing antibodies against both cytokines could decrease mortality [40].

Like the solTNFR1 profile, a high level of solTIM3 was observed in severely ill COVID-19 patients and those who died. However, it can be noted that it does not correlate with CRP. More studies are necessary to demonstrate if the high solTIM3 level is a consequence of the over-activation of ADAM17 and ADAM10, or whether it is a synergic effect of both metalloproteinases. Recently it was demonstrated that NK cells and CD8+ T cells from COVID-19 patients had overexpressed TIM3, associated with a hyperactivated/exhausted immune response dominant in severe SARS-CoV-2 infection [41].

It has been well documented that SARS-CoV-2 infects the target cell by interacting with the ACE2 receptor, and it is cleaved by ADAM17 and by transmembrane serine protease 2 (TMPRSS2) [42,43]. The increase in ADAM17 in the context of COVID-19 may have other implications that might be explored. For instance, ADAM17 promotes an increase in the pro-inflammatory response by activating signaling pathways such as the notch pathway, regulating proliferation, differentiation, and apoptosis [44,45]. Under viral infection contexts (influenza virus and human papilloma virus), ADAM17 attacks the cellular machinery and deregulates the notch pathway to improve the infection. Recently, it has been postulated that anti-TNF modulators increase the susceptibility to SARS-CoV-2 infection through Notch/IL-6 signaling via TMPRSS2-ADAM17 modulation in a macrophage model [45]. More evidence is required to clarify if this could be a generalized effect in patients with inflammatory diseases [46,47].

Relevant questions must be taken into consideration about the implications of the TNFR1/TNFR2 increment among COVID-19 subjects. Currently, it is still unclear if the elevated levels of TNFRs may represent an over-activation of ADAM17. One possibility is that the influence of SARS-CoV-2 is exerted directly on the receptors because research has reported that *Staphylococcus aureus* induces shedding of TNFR1 as an evasion mechanism of the immune response [48]. Another question is whether there are polymorphisms associated with TNF, TNFRs, ADAM17, and TIM3 in the context of SARS-CoV-2 infection. Under the SARS-CoV infection context, evidence has shown that single-nucleotide polymorphisms (SNPs) of the *TNF* promoter region (genotypes 1031CT/CC and 863) may represent risk factors in SARS [49].

This study also opens up several areas of investigation related to the effect of the high level of solTNFR1 in the serum of COVID-19 patients on the functions of the immunological cell, which probably occurs because this molecule activates a non-classical inflammatory mechanism that helps to develop the cytokine storm, and finally has negative consequences such as the presence of ARDS.

These data suggest that solTNFR1 and solADAM17 are increased in the severe condition of COVID-19, with a relationship to death. The field searching for therapy for COVID-19 is wide, with several open questions; as ADAM17 mediates activation of a vast number of proteins, we propose that solTNFR1 be considered a target in the development of new therapeutic immunomodulation for SARS-CoV2 infection.

## 4. Materials and Methods

### 4.1. Ethics Statement

The Ethical Research and Investigation Committees of the INER approved this protocol (#C41-20) as did the Investigation Committee of the HCM (#C.Inv.039). All individuals signed a consent letter to participate in this research, and procedures were performed following the 1964 Helsinki Declaration.

### 4.2. Study Design, Population and Samples Collection

Since 1 March 1 2020, the Mexican Health Ministry has dedicated some hospitals to exclusively attend to COVID-19 patients. The INER and HCM, among others, were designated as “exclusive COVID-19 hospitals”. INER is the leading institute for treatment of respiratory diseases in Mexico, and it is currently deployed almost exclusively as an intensive care unit. Therefore, most severe patients were recruited from the INER, whereas mildly and moderately ill patients were recruited mainly from HCM.

This work is a cross-sectional and descriptive study; it was carried out in 102 COVID-19 patients who tested positive for SARS-CoV-2 infection by RT-PCR of nasopharyngeal swabs. In addition, a control subject group of healthy donors (HD, *n* = 25) was included; HD subjects tested negative for SARS-CoV-2 infection by RT-PCR and were clinically evaluated by hospital staff. Blood samples of participants were obtained from INER and HCM in Mexico City, and the samples were gathered from May to September 2020.

COVID-19 patients were followed up by the clinician staff in the INER and HCM. The study population testing positive for SARS-CoV-2 showed at least 2 of the following symptoms: cough, fever, or headache, accompanied by at least 1 of the following signs or symptoms: difficulty breathing, anosmia, joint or muscle pain, conjunctivitis, throat pain, and nasal congestion. X-ray and/or tomography studies of the thorax in the initial assessment presented ground glass or bilateral consolidation opacities. Patients who did not have an X-ray and/or tomography study were excluded from this study. Other exclusion criteria were not having all the information of interest in the electronic files and not obtaining a blood sample.

This clinical data of the study were taken from the electronic files, structured records where sociodemographic, epidemiological, and clinical data as well as clinical laboratory test results from each patient were obtained.

### 4.3. Severity Classification of COVID-19

The hospitalized population in the HCM (53.9%) included mainly mild–moderate cases that did not require invasive mechanical ventilation and intensive therapy, while the INER mainly treated severe cases of ARDS and those that required intensive care (46.1%).

A severity scale for COVID-19 was reviewed in the institutional PACS system based on literature and clinical practice (CARE score, and National Institutes of Health COVID-19 Treatment Guidelines and Radiographic Assessment of Lung Edema score (RALE)), according to chest radiographs and tomography scans [50,51]. The results were reviewed by 3 pulmonologists and 1 thoracic radiologist physician. The referring unit for each examined radiography was documented and defined as follows: mild 1–2 points, moderate 3–6 points, and severe > 6. For this study, the severity scale used was previously assigned to each patient by the institutional clinical staff. This scale is in accordance with the WHO definitions [52] because, as observed in Table 2, oxygen saturation and respiratory rate are different among groups.

Consequently, COVID-19 patients were divided into 3 groups in concordance with the severity of the disease: 17 of them received a classification as mild, 31 as moderate, and 54 as severely ill. Finally, the fourth group (healthy donors, HD, *n* = 25) was included as a reference.

### 4.4. Serum and Peripheral Blood Mononuclear Cells Obtention

The blood samples were collected at hospital admission when the clinical laboratory staff took the blood sample for initial clinical analysis. The blood sample was obtained into a tube with clot activator and serum separator gel and a tube with spray-coated EDTA (BD Vacutainer 368159 and 367863, respectively, Franklin Lakes, NJ, USA). Sera were collected and kept at −20 °C until use; freezing–thawing processes were avoided. Peripheral blood mononuclear cells (PBMCs) were collected via a Ficoll density gradient (Lymphoprep Axis-Shield, Oslo, Norway); their viability was determined by using the Trypan Blue dye (Sigma-Aldrich, St. Louis, MO, USA) exclusion method. PBMCs were used for RNA and DNA extraction.

### 4.5. ELISA Sandwich Assays

TNF (Cat. No. 430201, BioLegend, San Diego, CA, USA), TNFR1, TNFR2, TGβ-1, and TIM3 (Cat. No. DY225, DY726, DY240, and DY2365 respectively; R&D Systems, Minneapolis, MN 55413, USA), and ADAM17 (Cat. No. SEB555Hu 96T, Cloud-Clone Corp., Katy, TX, USA) were evaluated. All the molecules were quantified by an ELISA assay in the sera, complying with the manufacturer’s instructions.

### 4.6. RNA Extraction and cDNA Synthesis

Total RNA was obtained from peripheral blood samples of HD and patients positive for SARS-CoV-2. Blood samples were stored at 70 °C in DNA/RNA Shield solution (Cat. No. R1100-250, Zymo Research, Irvine, CA, USA) to maintain RNA stability. Samples were diluted in a proportion of 1 to 3 volumes of solution, mixed, and then incubated at room temperature for cell lysis and inactivation of the coronavirus; finally, the homogenate was frozen. Before RNA extraction, samples were unfrozen, the RNeasy Micro Kit (Cat. No. 74106, Qiagen, Hilden, Germany) was used for RNA obtention, complying with the manufacturer’s instructions. Next, genomic DNA was removed using RNA-Free DNAse Set (Cat. No. 79254, Qiagen, Hilden, Germany), and RNA was eluted in 30 µL of nuclease-free water. The amount of RNA was evaluated by Qubit™ assay kit in the Qubit 2.0 Fluorometer (Life Technologies, Waltham, MA, USA). Finally, 135 ng of total RNA was used for first-strand cDNA synthesis using a High-Capacity cDNA Reverse Transcription Kit (Cat. No. 4368814, Applied Biosystems, Waltham, MA, USA) in a volume of 30 µL following manufacturer’s guidelines.

### 4.7. Real-Time Quantitative PCR

Real-time quantitative PCR (qRT-PCR) was performed using TaqMan probes specific for the target genes (Applied Biosystems, Waltham, USA): TNF (Hs00174128_m1), TNFR1 (Hs01042313_m1), TNFR2 (Hs00961750_m1), ADAM17 (Hs01041915_m1), ADAM10 (Hs00153853_m1), TGF-β (Hs00248373_m1), and TIM3 (Hs00958618_m1). 18S (18S ribosomal RNA gene) (Hs03928990_g1) and ACTB (β-actin) (Hs01060665_g1) were used as endogenous controls. Single reactions were prepared with the Maxima Probe/ROX qPCR Master Mix (No. Cat. K0231, Thermo Fisher Scientific, Waltham, USA), cDNA was diluted 1:4 (9 ng/µL to 2.25 ng/µL), and all amplifications were run by duplicate, under the following thermal conditions: 95 °C for 10 min followed by 40 cycles of 60 °C for 1 min and 95 °C for 15 s, with the StepOnePlus^TM^ Real-Time PCR Systems (Applied Biosystems, Waltham, MA, USA). The relative quantification of transcripts was quantified using the ΔΔCT method. The results were reported as the n-fold change for each target gene in each experimental group, normalized with the endogenous controls ACTB and 18S, and relative to a control group of ten HD (2^−ΔΔCT^ = 1).

### 4.8. Genetic Analysis

Genomic DNA was isolated from PBMC using the commercial (Cat. No. D3025, Quick-DNA^TM^ Miniprep Kit, Zymo Research BioAdvanced Systems, Irvine, CA, USA), and it was verified for purity, quality, and concentration before use. Six SNPs were evaluated; in *TNF* rs1800629 (probe assay: C___7514879_10) and 361525 (C___2215707_10), in *TNFRSF1A* rs767455 (C___2298465_20) and rs1800693 (C___2645714_10), and in *TNFRSF1B* rs1061622 (C___8861232_20) and rs3397 (C___8861228_20). All cases were genotyped by TaqMan SNP Genotyping assays (Applied Biosystems, San Francisco, CA, USA) in a 7300 Real-Time PCR System (Applied Biosystems/ThermoFisher Scientific Inc., Singapore). Contingency tables of 2 × 2 or 2 × 3 were used to compare the allele and genotype frequencies among groups using Epidat 3.1. The ClinVar accessions for the data were TNF SCV001548221.1, SCV001548220.1; *TNFRSF1A* SCV001548222.1, SCV001548223.1; and *TNFRSF1B* SCV001548218.1, SCV001548219.1.

### 4.9. Statistics

The Shapiro–Wilk normality test evaluated the data distribution, and indicated that our data did not have a normal distribution; therefore, data are reported as medians with interquartile ranges (25th and 75th percentiles, 95% CI). Comparisons between 2 groups were evaluated with the U Mann–Whitney test or Wilcoxon test, whereas multiple comparisons were performed with Kruskal–Wallis and corrected using Dunn’s test. Correlations between proteins in serum were determined with the Spearman correlation test. The survival curve (Kaplan–Meier) was compared using the log-rank test. Outlier exclusion was performed according to statistical identification by the Robust regression and Outlier removal method (ROUT), and it was applied to each evaluation. Values of *p* < 0.05 were considered statistically significant (GraphPad 7.0, Software, Inc., San Diego, CA, USA).

## 5. Conclusions

We concluded that solTNFR1 could be considered a target in the context of the development of immunomodulatory therapy. Although ADAM17 was also increased, the blocking of ADAM17 could have severe consequences since it is necessary to mediate the delivery of many other proteins. Thus, our data support the concept of using anti-TNF therapy in COVID-19, as has been suggested by observational clinical data [53,54]. However, we suggest that the measure of TNF, TNFR1, and TNFR2 be performed on a per-patient basis to establish a better individual scheme of treatment.

## Figures and Tables

**Table 1:** Demographic and comorbid state.

	Healthy Donor (HD) *n* = 25	Mild *n* = 17	Moderate *n* = 31	Severe *n* = 54	*p*-Value
Age (y)	33 ± 7	44 ± 13	53 ± 14	51 ± 14	<0.0001 ^a,b^
Gender (M:F)	7:18	10:7	21:10	39:15	<0.01 ^a,b^
Smoking	0	0	2 (6%)	12 (22%)	<0.05 ^b^
Diabetes mellitus	0	1 (6%)	1 (3%)	13 (24%)	<0.01 ^b^
Systemic Hypertension	0	1 (6%)	3 (10%)	6 (11%)	nd
Body mass index (kg/m^2^)	26 ± 3	30 ± 4	31 ± 7	31 ± 7	<0.01 ^b,c^
HCM/INER ^¥^	18/7	17/0	23/8	17/37	na

Age and body mass index are represented as means ± SD. Comorbidities are represented as *n* (%). ^¥^ Ratio of the blood samples provided by Central Military Hospital (HCM) and National Institute of Respiratory Diseases (INER). ^a^ Differences among HD and moderate groups; ^b^ Differences between HD and severe groups; ^c^ Differences between HD and mild groups. na: not applicable. nd: no differences.

**Table 2:** Symptoms and signs presented in COVID-19 patients.

Symptom	Mild *n* = 17	Moderate *n* = 31	Severe *n* = 54	*p*-Value
Fever	10 (59%)	19 (61%)	32 (59%)	nd
Cough	10 (59%)	16 (52%)	32 (59%)	nd
Dyspnea	2 (11%)	18 (58%)	4 (7%)	<0.01 ^a,b^ <0.0001 ^a,c^
Diarrhea	2 (11%)	2 (6%)	7 (13)	nd
Anosmia	0	2 (6%)	5 (9%)	nd
Fatigue	6 (35%)	14 (45%)	31 (57%)	nd
Headache	4 (24%)	8 (26%)	19 (35%)	nd
Heart rate ^£^	91 ± 10	106 ± 19	106 ± 19	nd
Breathing frequency ^£^	20 ± 2	22 ± 5	27 ± 8	<0.01 ^a,c^ < 0.01 ^b,c^
Oxygen saturation ^£^	92 ± 4	86 ± 13	71 ± 19	<0.0001 ^a,c^ <0.01 ^b,c^

Symptoms are represented as *n* (%, presence). ^£^ Data are presented as *n* or mean ± SD. Heart rate (BPM, beats/min), breathing frequency (breaths/min), oxygen saturation (%). ^a^ Mild; ^b^ Moderate; ^c^ Severe. nd: no differences.

**Table 3:** Clinical parameters measured at the time of institutional admission.

Parameter	Mild	Moderate	Severe
Leucocytes (RV = 4–10 × 10^3^ cells/mm^3^)	8.2 ± 3.9	9.7 ± 4.8	11 ± 6.1
Lymphocytes (RV = 1–4 × 10^3^ cells/mm^3^)	1.8 ± 1.6	2.2 ± 4.1	1.1 ± 1
C-reactive protein (RV = 1–10 mg/dL)	81 ±71	81 ±71	993 ±57
Fibrinogen (RV = 200–400 mg/dL)	537 ± 224	565 ± 245	686 ± 223
Glucose (RV = 74–106 mg/dL)	162 ± 115	176 ± 111	169 ± 99

RV = Reference value (provided by the institutional clinical laboratory). Values are indicated as mean ± SD. No differences were found between groups.

## Data Availability

All data relevant to the study are included in the article or uploaded as supplementary information. Gene variant data has been submitted to ClinVar (SCV001548218, SCV001548219, SCV001548220, SCV001548221, SCV001548222, SCV001548223). Authors confirm that the raw data to support the conclusions of this study are included in the manuscript. The corresponding author will provide more information, upon rational request, to any qualified researcher. The datasets presented in this study can be found in online repositories. The names of the repository/repositories and accession number(s) can be found in the Materials and Methods Section 4.8 Genetic Analysis.

## Supplementary Materials

The following are available online at https://www.mdpi.com/article/10.3390/ijms22168423/s1.

## Abbreviations

ACE2	Angiotensin-converting enzyme-2
ADAM10	A disintegrin and metalloproteinase domain-containing protein 10
ADAM17	A disintegrin and metalloprotease 17 (also called TACE)
ARDS	Acute respiratory distress syndrome
COVID-19	Coronavirus disease 2019
CRP	C-reactive protein
ELISA	Enzyme-linked immunosorbent assay
HD	Healthy donors
HCM	Central Military Hospital
HMGB1	High mobility group box 1
IL	Interleukin
INER	National Institute of Respiratory Diseases
PBMC	Peripheral blood mononuclear cells
qRT-PCR	Real-time reverse transcription polymerase chain reaction assay
ROUT	Robust regression and outlier removal method
RT-PCR	Real-time reverse transcriptase
SARS-CoV-2	Severe acute respiratory syndrome coronavirus 2
solTGF-β	Soluble TGF-β
solTIM3	Soluble TIM3
solTNF	Soluble TNF
solTNFR1	Soluble TNFR1
solTNFR2	Soluble TNFR2
SNPs	Single-nucleotide polymorphisms
TACE	TNF-α converting enzyme (also called ADAM17)
TGF-β	Transforming growth factor-beta
TIM3	T cell immunoglobulin and mucin domain 3
TMPRSS2	Transmembrane protease serine 2
tmTNF	Transmembrane TNF
TNF	Tumor necrosis factor
TNFR1	TNF receptor 1
TNFR2	TNF receptor 2
