# Supplementary material for: Severe COVID-19 Patients Show an Increase in Soluble TNFR1 and ADAM17, with a Relationship to Mortality
Source: Int J Mol Sci. 2021 Aug 5;22(16):8423. doi: 10.3390/ijms22168423 (PMC8395100; doi:10.3390/ijms22168423)
Supplement: Supplementary file 1 [file ijms-22-08423-s001.zip › ijms-1288368-supplementary.pdf]

Supplementary Materials

Table S1. Allele and genotype frequencies of the variants included in the study (*n* = 80).

Gene/ Variant	Allele/ Genotype	Control *n*=18 (%)	Mild *n*=18 (%)	Moderate *n*=22 (%)	Severe *n*=22 (%)
TNFRSF1A					
	T	23 (63.9)	27 (75.0)	32 (72.1)	24 (54.5)
	C	13 (36.1)	**9 (25.0)**	12 (27.3)	**20 (45.5)**[a]
rs767455	TT	8 (44.4)	10 (55.6)	11 (50.0)	6 (27.3)
	TC	7 (38.9)	**7 (38.9)**	10 (45.5)	**12 (54.5)**[b]
	CC	3 (16.7)	**1 (5.6)**	1 (4.5)	**4 (18.2)**[c]
	T	25 (69.5)	27 (75.0)	33 (75.0)	27 (61.3)
	C	11 (30.5)	9 (25.0)	11 (25.0)	17 (38.7)
rs1800693	TT	8 (44.5)	10 (55.5)	11 (50.0)	6 (27.3)
	TC	9 (50.0)	7 (38.9)	11 (50.0)	15 (68.2)
	CC	1 (5.5)	1 (5.5)	0	1 (4.5)
TNFRSF1B					
	T	30 (83.3)	35 (97.2)	39 (88.6)	40 (90.9)
	G	6 (16.7)	1 (2.8)	5 (11.4)	4 (9.1)
rs1061622	TT	12 (66.7)	17 (94.4)	18 (81.8)	18 (81.8)
	TG	6 (33.3)	1 (5.6)	3 (13.6)	4 (18.2)
	GG	0	0	1 (4.5)	0
	T	29 (80.6)	27 (75.0)	37 (84.1)	40 (90.9)
	C	7 (19.4)	9 (25.0)	7 (15.9)	4 (9.1)
rs3397	TT	12 (66.7)	11 (61.1)	15 (68.2)	18 (81.8)
	TC	5 (27.8)	5 (27.8)	7 (31.8)	4 (18.2)
	CC	1 (5.6)	2 (11.1)	0	0
TNF					
	G	36 (100.0)	36 (100)	42 (95.5)	41 (93.2)
	A	0	0	2 (4.5)	3 (6.8)
rs1800629	GG	18 (100.0)	18 (100)	20 (90.9)	19 (86.4)
	GA	0	0	2 (9.1)	3 (13.6)
	AA	0	0	0	0
	G	36 (100.0)	34 (94.4)	42 (95.5)	43 (97.7)
	A	0	2 (5.6)	2 (4.5)	1 (2.3)
rs361525	GG	18 (100.0)	16 (88.9)	21 (95.5)	21 (95.5)
	GA	0	2 (11.1)	1 (4.5)	1 (4.5)
	AA	0	0	0	0

Marginal associations are highlighted in bold style. [a] p = 0.0583, OR = 2.5(0.95–6.52); [b] p = 0.0580, OR = 2.8(0.72–11.31); [c] p = 0.0580, OR = 6.6(0.59–74.50).

Table S2. Dominant and recessive model analyses for the variants included in the study (n = 80).

Variant	Dominant model	Control n=18 (%)	Mild n=18 (%)	Moderate n=22 (%)	Severe n=22 (%)
rs767455	TT	8 (44.4)	10 (55.6)	11 (50.0)	6 (27.3)
	TC+CC	10 (55.6)	**8 (44.4)**	11 (50.0)	**16 (72.7)**[a]
rs1800693	TT	8 (44.4)	10 (55.6)	11 (50.0)	6 (27.3)
	TC+CC	10 (55.6)	**8 (44.4)**	11 (50.0)	**16 (72.7)**[a]
rs1061622	TT	12 (66.7)	17 (94.4)	18 (81.8)	18 (81.8)
	TG+GG	6 (33.3)	1 (5.6)	4 (18.2)	4 (18.2)
rs1800629	GG	18 (100.0)	18 (100)	20 (90.9)	19 (86.4)
	GA+AA	0	0	2 (9.1)	3 (13.6)
rs361525	GG	18 (100.0)	16 (88.9)	21 (95.5)	21 (95.5)
	GA+AA	0	2 (11.1)	1 (4.5)	1 (4.5)
rs3397	TT	12 (66.7)	11 (61.1)	15 (68.2)	18 (81.8)
	TC+CC	6 (33.3)	7 (38.9)	7 (31.8)	4 (18.2)
**Variant**	**Recessive model**	**Control n=18 (%)**	**Mild n=18 (%)**	**Moderate n=22 (%)**	**Severe n=22 (%)**
rs767455	TT+TC	15 (83.3)	17 (94.4)	21 (95.4)	18 (81.8)
	CC	3 (16.7)	1 (5.6)	1 (4.5)	4 (18.2)

Marginal associations are highlighted in bold style. [a]p = 0.069, OR = 3.3(0.9–12.5).
